# Structural basis of cholesterol binding by a novel clade of dendritic cell modulators from ticks

**DOI:** 10.1038/s41598-017-16413-2

**Published:** 2017-11-22

**Authors:** Pietro Roversi, Steven Johnson, Stephen G. Preston, Miles A. Nunn, Guido C. Paesen, Jonathan M. Austyn, Patricia A. Nuttall, Susan M. Lea

**Affiliations:** 1 Biochemistry Department, University of Oxford, Oxford, OX1 3QU England United Kingdom; 2 Sir William Dunn School of Pathology, University of Oxford, Oxford, OX1 3RE England United Kingdom; 3 Department of Zoology, University of Oxford, Oxford, OX1 3PS England United Kingdom; 4 Akari Therapeutics, Plc, 75/76 Wimpole Street, London, W1G 9RT England United Kingdom; 5 Division of Structural Biology, Wellcome Trust Centre for Human Genetics, University of Oxford, Oxford, OX3 7BN United Kingdom; 6 Nuffield Department of Surgical Sciences, John Radcliffe Hospital, University of Oxford, Oxford, OX3 9DU England United Kingdom; 7 Leicester Institute of Structural and Chemical Biology, Department of Molecular and Cell Biology, University of Leicester, Henry Wellcome Building, Lancaster Road, Leicester, LE1 7RH England United Kingdom

## Abstract

Two crystal structures of Japanin, an 18 kDa immune-modulatory lipocalin from the Brown Ear Tick (*Rhipicephalus appendiculatus*), have been determined at 2.2 and 2.4 Å resolution. In both crystal forms the protein is in complex with cholesterol, which sits in a closed pocket at the centre of the lipocalin barrel. Both crystal forms are dimers, which are also observed in solution. Molecular modelling suggests that previously-described members of a tick protein family bearing high sequence homology to Japanin are also likely to bind cholesterol or cholesterol derivatives.

## Introduction

Hard ticks are obligate haematophagous parasites with an unusually lengthy feeding period for an ectoparasite (up to 15 days), during which the tick modulates the host immune response to prevent it mounting an effective anti-parasite response. This immunomodulation is mediated by a complex cocktail of compounds, affecting several arms of the immune response^1^, including the activation of dendritic cells (DC), a central process in the initiation of adaptive immunity. For example, unfractionated saliva from *Rhipicephalus sanguineus* inhibits dendritic cell maturation and differentiation^2^; Salp15, an *Ixodes scapularis* salivary gland protein, inhibits dendritic cell secretion of pro-inflammatory cytokines^3^; while Japanin, an 18 kDa protein recently isolated from the salivary glands of a hard tick, *Rhipicephalus appendiculatus* (the Brown Ear Tick), works through currently undefined mechanisms to block DC differentiation from monocytes and inhibits upregulation of co-stimulatory molecules and pro-inflammatory cytokines in response to stimuli. Japanin also promotes upregulation of co-inhibitory molecules and the anti-inflammatory cytokine interleukin-10^4^.

Japanin is predicted from sequence data to be a lipocalin, a member of a family of ubiquitous small proteins found in both prokaryotes and eukaryotes. Lipocalins are characterised by the presence of an 8-stranded beta barrel structure which typically sequesters a small hydrophobic ligand^5^. In ticks, the lipocalin family shows a large expansion, with many lipocalins detected in the salivary gland transcriptome and appearing to bind biogenic amines such as histamine, or fatty acids such as leukotrienes, helping control inflammation and aiding blood-feeding^6–8^.

To provide insight into Japanin’s mechanism of action, we here present two crystal structures of the protein. They reveal that Japanin exists in complex with cholesterol and that it forms a dimer, as well as confirming the prediction from primary sequence that it adopts the lipocalin fold^9^. Japanin thus becomes the first lipocalin for which the molecular details of cholesterol binding are described.

## Results

### Crystal structures

**Figure 1:**
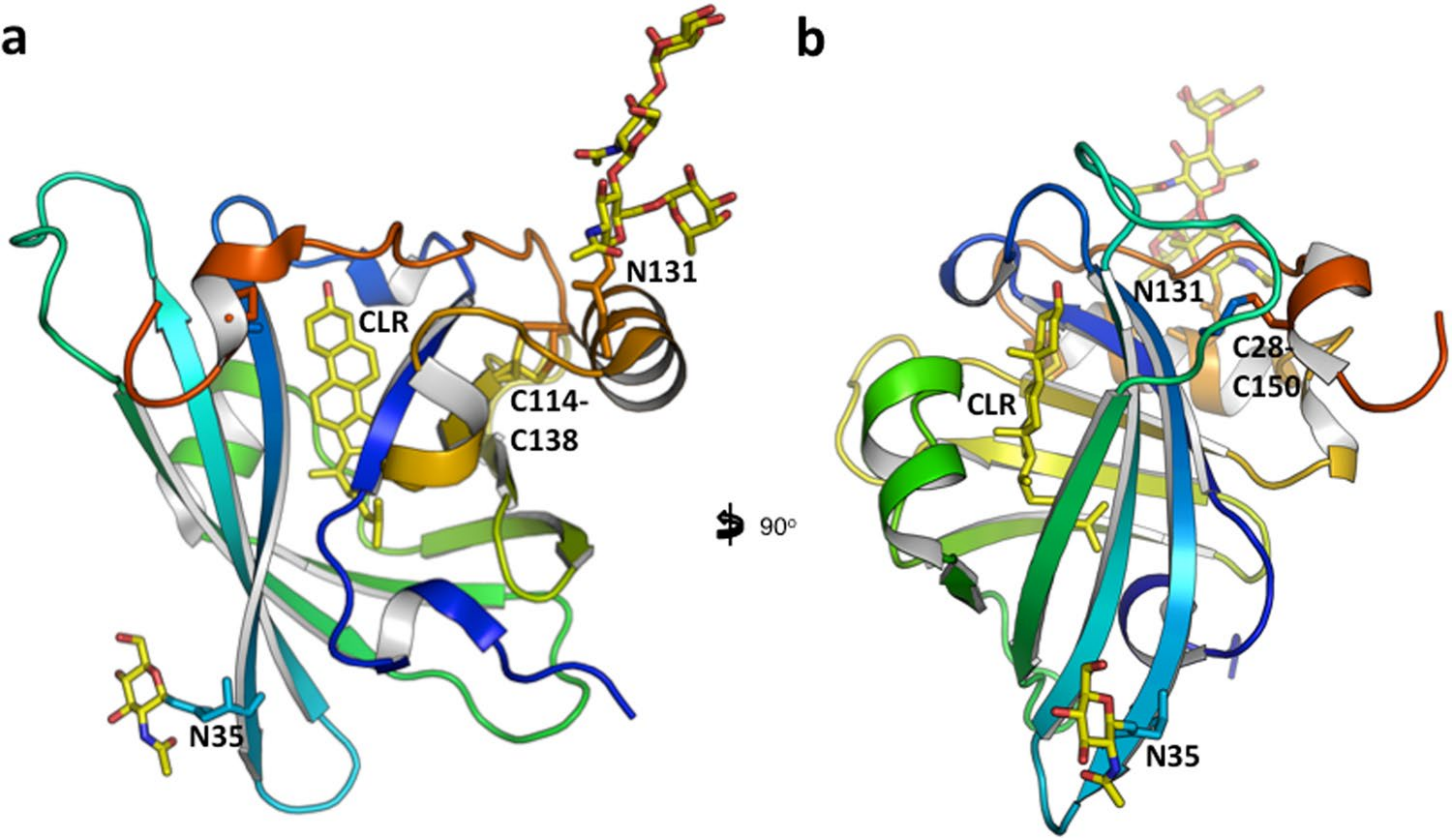
Japanin monomer. The *Rhipicephalus appendiculatus* Japanin monomer from the tetragonal crystal form is in cartoon representation, coloured blue to red from N- to C-terminus. The views in (**a**) and (**b**) differ by a rotation of 90° around the vertical axis. The Cys28-Cys150 and Cys114-Cys138 disulphide bonds, Asn35, Asn131 and their N-linked glycans, and the bound cholesterol molecule are in sticks representation. Pictures prepared with PyMOL.

We obtained two crystal structures of recombinantly expressed *R. appendiculatus* Japanin: (i) a tetragonal form with one copy per asymmetric unit (data to 2.2 Å) and (ii) an orthorhombic one with two molecules per asymmetric unit (data to 2.4 Å), both in complex with cholesterol. All three crystallographically-independent molecules show the same overall structure, and each molecule in the crystals binds one molecule of cholesterol. It is noted that no cholesterol was added exogenously at any stage during purification or crystallisation. As was predicted from the sequence, the protein folds as a lipocalin, with an 8-stranded anti-parallel barrel at its centre. The three crystallographically-independent molecules superimpose with an overall C_α_ rmsd of 1.3 Å across 152 residues (overlap computed with the program Theseus^10^). The main sites of conformational mobility are the hairpin loop 48–58, the loop 105–112 and the C-terminus, residues 145–152. A search against the Protein Databank reveals that the closest structural homologues are the female-specific histamine-binding protein (FS-HBP2; PDB IDs 3g7x, 1qft) with a rmsd C_α_ of 2.7 Å over 133 residues; and the OmCI complement inhibitor (PDB IDs 2cm4, 2cm9, 3zuo, 3zui, 5hcc, 5hcd, 5hce) with a rmsd C_α_ of 2.5 Å over 123 residues^11^. Two disulphide bonds are observed (Japanin residues Cys28-Cys150 and Cys114-Cys138), the latter taking two alternative conformations. Residues Asn35 and Asn131 bear N-linked glycans, but they are not in close proximity, neither within the monomer nor in the context of the dimer^12^. Figure 1 shows two views of the protein.

### Japanin dimer

**Figure 2:**
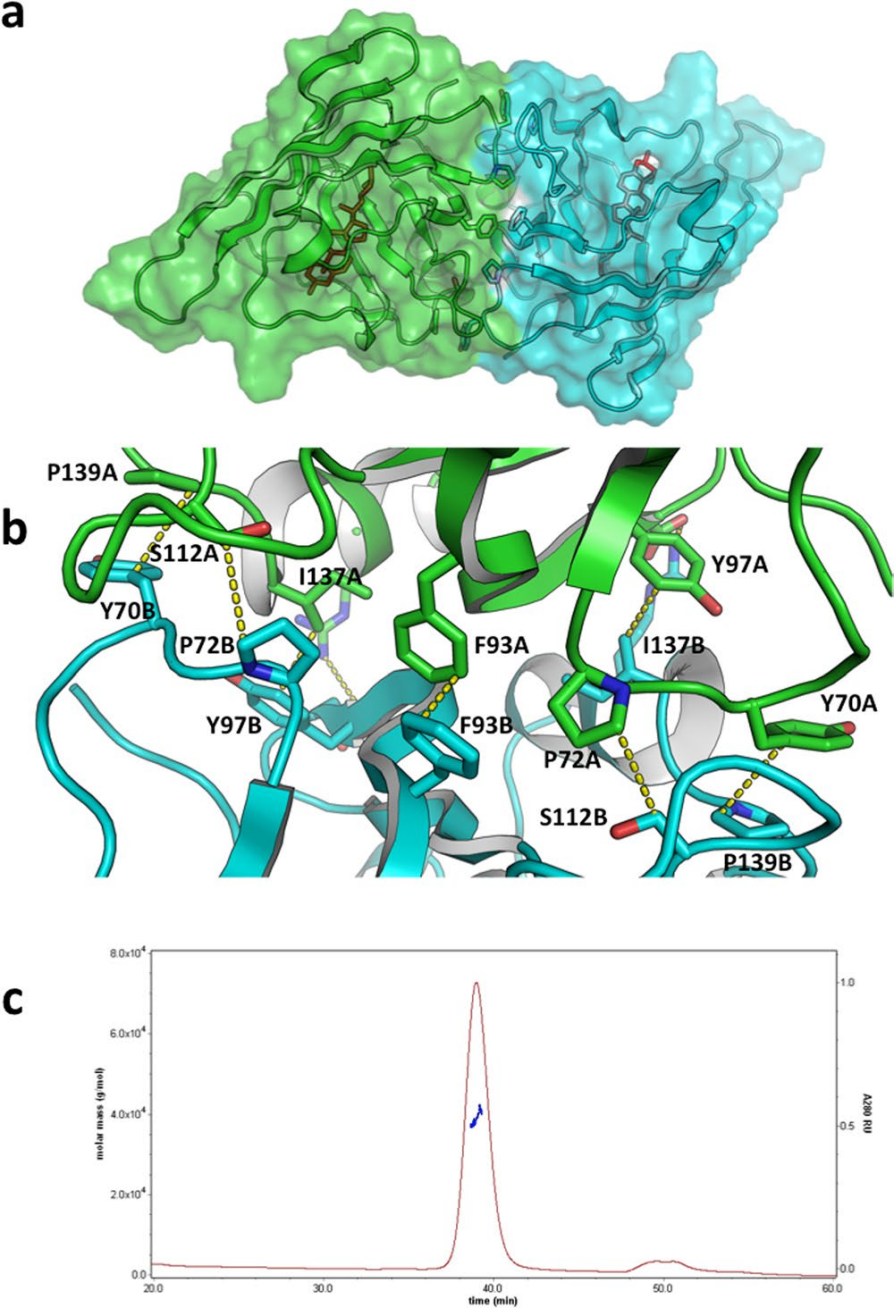
Japanin dimer. (**a**) The *Rhipicephalus appendiculatus* Japanin dimer from the orthorhombic crystal form (coloured green and cyan for chains A and B respectively). The cholesterol molecules are represented by red sticks. (**b**) Details of the dimer interface. Carbon atoms of molecule B in cyan, carbon atoms of molecule A in green. Oxygen red, nitrogen blue. H atoms omitted. Close contacts are reported in yellow dotted lines. (**c**) SEC-MALLS analysis of the recombinant protein. The sample was run on a Superdex 200 (10/300) column at 0.4 ml/min. The measured mass across the elution peak is shown as a blue line and corresponds to a dimer. Pictures prepared with PyMOL.

Both crystal forms contain the same Japanin dimer, which has an interface area of about 1090 Å^2^, involving 32 residues and a calculated solvation free energy gain upon formation of the interface of −9.5 kcal/mole (as computed with the protein interfaces, surfaces and assemblies’ service PISA at the European Bioinformatics Institute^13^). In the orthorhombic form, the two molecules in the asymmetric unit form the dimer (see Fig. 2a). In the tetragonal crystals, the same dimer is formed by the asymmetric unit and a symmetry-related molecule across a twofold axis. The tetragonal crystal-form dimer and the orthorhombic crystal-form dimer superpose with an rmsd of 1.5 Å over 297 C_α_s.

The dimer interface involves the 67–75 and 105–112 loops, the 93–99 strand and the 129–141 helix. Figure 2b shows details of the dimer interface between molecules A and B in the lattice, centred around the Phe93A:Phe93B side chain stacking contact, with two additional pairs of hydrophobic residues forming contacts across the interface: Pro139A:Tyr70B and Tyr97A:Ile137B (and equivalent ones due to the twofold symmetry of the dimer, Pro139B:Tyr70A and Tyr97B:Ile137A). Two additional hydrophobic contacts are formed between CH2 moieties of Ser112 and Pro72 (again intermolecularly). Buried in the middle of this hydrophobic patch are the 2.0 Å hydrogen bonds between the side chain of Arg95 on one molecule and the main chain oxygen atom of Ile 94 on the neighbouring molecule. At the surface rim of the interface the Asp98A:Arg132B (and Arg132A:Asp98B) salt bridges are found. The dimer is likely to represent the solution species, as indicated by Multi-Angle Laser Light Scattering (MALLS) in solution (see Fig. 2c).

### Cholesterol binding

**Figure 3:**
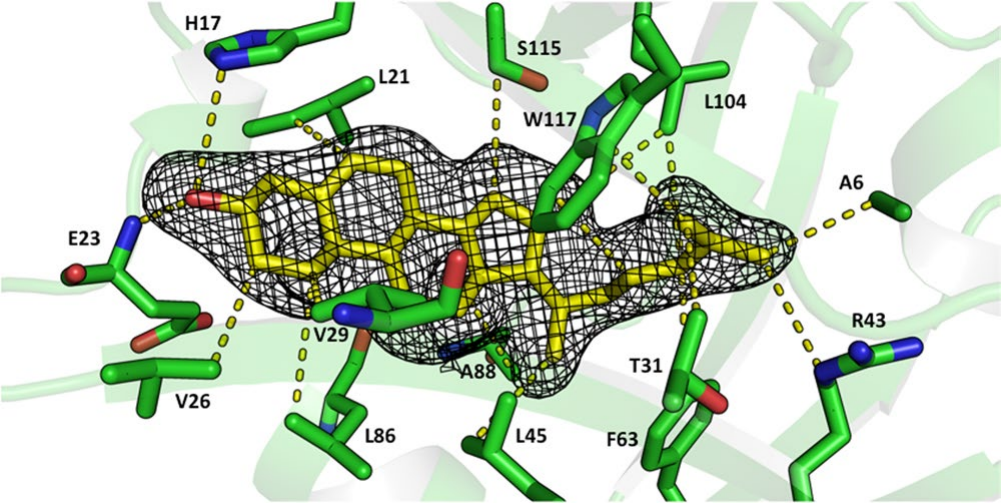
Cholesterol binding in the Japanin pocket. The Japanin cholesterol binding pocket and cholesterol ligand from PDB ID 4boe. C atoms of cholesterol are coloured yellow. Japanin side chains contacting the ligand are in sticks representation and the contacts between cholesterol and the protein are depicted in yellow dashed lines. The 3σ level contour of an Fo-Fc difference density map, computed before modelling the ligand, is represented as a mesh in the region of the cholesterol ligand. Picture prepared with PyMOL.

In each of the three crystallographically-independent molecules, fifteen residues lining the lipocalin pocket are involved in close intermolecular contacts with a cholesterol molecule, with a ligand:protein interaction surface of about 280 Å^2^, 3% of the surface of the molecule, see Fig. 3. Residues Ala6, Leu21, Val26, Val29, Thr31, Arg43, Leu45, Phe63, Leu86, Ala88, Leu104, Ser115 and Trp117 all make hydrophobic contacts shorter than 3.5 Å with the cholesterol molecule; the -OH group of cholesterol hydrogen bonds with the main chain NH of Glu23 (distance O-H: 2.0 Å) and to the Nε_***2***_ atom of His17.

**Figure 4:**
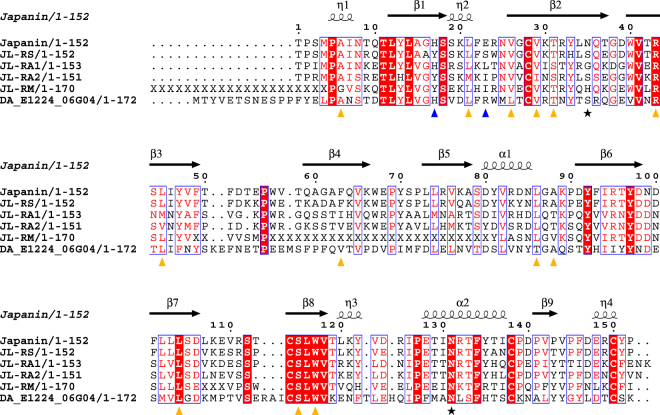
Japanin sequence and secondary structure aligned to its sequence homologues. DA: *Dermacentor andersoni*. RM: *Rhipicephalus* (*Boophilus*) *microplus*, RS: *R. sanguineus*, RA: *R. appendiculatus*. Red: conserved residues. η: 3_*10*_ helix. Red character on white: Risler Similarity Score (Risler, 1988) greater than 0.7. Yellow triangles: residues making hydrophobic contacts to the cholesterol molecule (distances lower than 3.5 Å). Blue triangles: residues hydrogen bonds to the cholesterol molecule. Stars: glycosylation sites. Disulphide bonds are observed between Japanin residues Cys28-Cys150 and Cys114-Cys138.

The presence of cholesterol in association with Japanin raises obvious questions as to the functional role of cholesterol binding to the protein: in particular, whether the presence of cholesterol in the lipocalin pocket is related to Japanin’s immune-modulatory function. Cholesterol and a range of cholesterol derivatives have been shown to have immune-modulatory roles, either directly^14–16^ or through interactions with the gut microbiota^17^. Modelling of cholesterol derivatives in the Japanin ligand-binding pocket shows that epicholesterol, epicholestanol, epicoprostanol, and 7-dehydrocholesterol could fit in the pocket with minor rearrangements of the side chains. A 22(R)-hydroxy group would lead to a clash with Val76, making binding to derivatives carrying this group unlikely. Residues Thr31, Arg43, Ser115 and Trp117 could hydrogen bond to cholesterol derivatives bearing polar groups on the tail, such as 25-OH-cholesterol. Amino-acid side chains that carry a charge when on the surface of proteins at physiological pH are the exception rather than the rule within apolar protein pockets, and yet the Japanin pocket features Arg43 (completely conserved across members of the clade (Fig. 4)) and His17, whose Nε_***2***_ atom is only 3.9 Å from the ligand’s 3-hydroxy group. Thanks to the latter residue, cholesterol derivatives carrying a negative charge on the 3-hydroxy group (*e.g*. cholesterol sulfate) may therefore bind to the Japanin pocket more tightly than cholesterol. The Arg43 guanidinium side chain, on the other hand is buried at the base of the pocket and involved in a hydrogen bonding network with the main chain O atoms of Ala6, Asn8, Gln10, Thr31 and the hydroxyl group of the latter residue. It is possible that the Arg43 side chain could engage cholesterol derivatives carrying polar groups on the tail, such as 27-hydroxycholesterol^18^.

### Japanin homologues are likely to bind cholesterol or cholesterol-derivatives

A number of tick lipocalins have sequences that are highly similar to that of Japanin, forming a clade of tick dendritic cell modulators^4^. Figure 4 shows the sequence alignment of the Japanin protein to those homologues, annotated with the secondary structure elements and painted by conservation. The high degree of sequence conservation of the residues involved in the dimer interface suggests these proteins would also dimerise like Japanin. Based on sequence conservation and homology modelling, RA1, RA2 and RS are also all as likely to bind cholesterol as Japanin (no clashes in the pocket). Furthermore, the *Dermacentor andersoni* putative protein DA1244 would also be capable of binding cholesterol (most of the pocket is very conserved) and in fact could accommodate a larger ligand due to the substitution of Japanin F63 for DA1244 V66.

## Discussion

Cholesterol and its derivatives, besides being major components of eukaryotic cellular membranes, play essential roles in cellular functions as important and as varied as intracellular transport; cell signalling; nerve conduction; regulation of lipid, glucose, and energy metabolism^19^; drug metabolism and detoxification; and inflammation^20,21^. For example, cholesterol binding is crucial for human GPCR Smoothened to transmit native Hedgehog signalling^22,23^.

Tear lipocalins^24^ and proteins belonging to different fold families have been reported to bind and either transport or chemically modify cholesterol^25–27^. Table 1 reports cholesterol-binding protein domains whose crystal structures were determined in their apo forms or in complex with either cholesterol or cholesterol derivatives. In the Japanin crystals, the cholesterol ligand is completely sequestered inside the protein, except for the –OH group at position 3, which points towards a solvent accessible cavity. This is similar to what was observed for the cholesterol molecules in the crystal structures of yeast Osh4^28^; in the ligand binding domain of the nuclear receptor ROR; and in the human cytochrome CYP11A1^29^. The crystal structure of the human cytochrome CYP46A1^30^ shows a cholesterol sulphate molecule fully trapped inside the protein. The crystal structures of the cholesterol-binding steroidogenic acute regulatory protein transport (START) domain proteins MLN64 and StarD4 also showed fully closed pockets, although no ligands were present in those crystals^31–33^. The cholesterol-binding domains of proteins Niemann-Pick C1 and C2^34–36^ and proteins of the Scp2 family^37–40^ have similar ligand-binding tunnels, although some of these are open at one end. In most cholesterol binding proteins of known structure, a conformational change would therefore be required for uptake and release of the ligand. Opening and closing of a protein “lid” to favour exchange of cholesterol has been invoked for example for the oomycete protein cryptogein^41^. The Japanin loop ^*18*^SSKLFERNVG^27^ is not kept in place by any strong bonds, and might therefore form an opening and closing lid, although its thermal motion in the crystals is not particularly higher than the rest of the structure.

**Table 1:** Cholesterol-binding proteins of known structure.

	Japanin	Osh4	ROR	Cryptogein	NPC1,NPC2	CYP11A1, CYP46A1	CE, type I 17 β-HSD	Smoothened
**Organism**	*Rhipicephalus appendiculatus*	Yeast	*Homo sapiens*	*Phytophthora cryptogea*	*Homo sapiens*	*Homo sapiens*	*Candida cylindracea*	*Homo sapiens*
**PDB ID**	4boe	1zhy	1n83,1s0x	1lri	3gki,2hka	3n9y, 2q9f	1cle, 1llf	5l7d
**Ligand**	CLR	CLR	CLR,CLR-SO_***4***_	CLR				CLR
**Lid**	^***18***^SSLFERLMG^27^	^22^GDLS^25^		^32^GYSMLTAKALPT^43^ and ^75^VPTSGL^80^				^155^AIVERERGWPDFLR^168^
**3-OH binding**	H17	Q96	R370	Y47				D95
**Reference**	This work	^28^	50,62	^41^	^34,36^	29,30	^25,26^	^22,23^

Biophysical simulations using the Protein Energy Landscape Exploration (PELE) algorithm revealed low binding energies for cholesterol, typical of a ligand^42^. However, *in silico* docking suggests that the Japanin pocket can accommodate a variety of cholesterol derivatives. Japanin’s natural ligand(s) may therefore be cholesterol derivatives which - unlike cholesterol - were not available during recombinant protein production. It is also possible that a ligand present when Japanin is produced by the tick may be exchanged for a cholesterol derivative following secretion into the host. Indeed, arthropods are unable to synthesize cholesterol *de novo* ^43^, so it is likely that a cholesterol ligand would be bloodmeal-derived. Moreover, uptake of Japanin by the host endocytic pathway would result in a low pH environment following endosomal acidification, perhaps promoting intracellular ligand release in favour of a cholesterol derivative with a negative charge on a moiety bound to the 3-hydroxy group. Alternatively, going from alkaline tick saliva into serum might be enough to trigger an exchange, especially at inflammatory sites, where pH is known to drop^44^. The presence of Arg43 and His17 in the binding pocket may point to pH changes as critical to ligand selectivity and to the ligand binding/releasing process. Inside the pocket, without neighbouring acidic amino-acids side chains, the pK_A_ of the guanidinium side chain of Arg43 and His17 may differ from the ones for the same amino acids in isolation. Their side chains may be uncharged at physiological pH, when the molecule is empty or cholesterol is in the pocket. Alternatively, a polar (charged) cholesterol derivative may be the physiological ligand, and a hydrogen bond (salt bridge) between a Arg43 and/or His17 and a polar (charged) cholesterol derivative would ensure permanent trapping of the ligand after sequestration. Last but not least, Japanin residues Thr31, Arg43, Ser115 and Trp117 could hydrogen bond to cholesterol derivatives bearing polar groups on the tail: oxidized derivatives of cholesterol are potent immune-suppressors, with 25-hydroxycholesterol for example acting as an inhibitor of humoral and cellular responses^45^. The most relevant of these compounds (given the role of Japanin in dendritic cell biology) is 7-alpha, 25-dihydroxycholesterol (7,25,DHC), which functions as a guidance cue for EBI2-expressing dendritic cells, positioning them in a location where they encounter blood-borne particulate antigens^46^. Overlaying 7,25,DHC onto the cholesterol molecule in the crystal structure places the 7-hydroxyl group within hydrogen bonding distance of Serine 115, a residue in the completely conserved ^114^CSLWV^118^ motif in the Japanin clade (see the alignment in Fig. 4).

Whatever the physiological ligand(s), lipids play important roles in the metabolism and activation of immune cells^47^, and it is plausible that Japanin’s function is mediated by ligand sequestration. For example, a recent study discovered that cholesterol crystals activate Syk and PI3 kinases in human macrophages and dendritic cells, driving IL-1 production in a Syk- and PI3K-dependent manner, and activating the downstream MAP kinases; in these human innate immunity cells, cholesterol induces S100 and MMP1 expression via tyrosine kinases^48^. Olesoxime, a cholesterol-like compound, has been shown to favor oligodendrocyte maturation in culture and promote myelin regeneration in rodents^49^. The effects of cholesterol-deprivation on immunity are suggested by the phenotype of Staggerer mice which are defective in the cholesterol-binding ROR nuclear receptor and exhibit defects in the immune and inflammatory response^50^. Collectively these observations suggest that cholesterol sequestration by Japanin, perhaps localised at the bite site, might be directly responsible for at least some of Japanin’s immune-modulatory properties.

In summary, the Japanin’s crystal structures have revealed it to be the prototypic cholesterol-binding lipocalin, a group which is very likely to include other members of the Japanin-like clade of hard tick lipocalins, while also hinting at directions for future research into the mechanism of action of this unusual immune-modulatory molecule.

## Materials and Methods

### Protein expression and purification

Protein expression and purification was as previously described^4^. In brief, polyhistidine-tagged recombinant protein was produced by Sf9 cells infected with recombinant baculovirus, then purified using Talon resin followed by gel filtration.

### Crystallisation, X-ray diffraction and data processing

Crystals were grown in the course of several weeks by the vapour diffusion method in sitting drops at 21 °C, set up using an Oryx nano crystallisation robot (Douglas, UK).

The crystallisation drops were set up by mixing 0.12 μl of protein solution at OD_***280***_ = 6.6 (approximately 5.2 mg/ml) in gel filtration buffer (50 mM Tris.HCl, pH 7.5, 150 mM NaCl) with 0.08 μl of the crystallisation screen, and were equilibrated against 70 μl of mother liquor.

Japanin-P 4_***1***_2_***1***_2: square-bipyramidal crystals initially grew from condition H9 of the Molecular Dimensions ProPlex screen: 0.1 M imidazole pH 7.0 and 50% v/v 1-methyl-pentan-(2,4)diol (MPD). The crystals could be reproduced by screening around the condition and grew in the pH interval 6.9–7.2 and MPD range 40–50%. A 2.2 A X-ray diffraction dataset was collected from one such crystal and indexed in a tetragonal primitive space group. Scaling and systematic extinctions suggested P4_***1***_2_***1***_2 or P4_***3***_2_***1***_2 (details in Table 2).

**Table 2:** X-ray data collection statistics.

PDB ID	4boe	4bqu
Space Group (Z)	P 4_***1***_2_***1***_2 (8)	C222_***1***_ (16)
X-ray source	ESRF ID29	ESRF ID29
Detector	ADSC	ADSC
**Wavelength (Å)**	0.96	0.9763
a (Å)	84.33	80.18
b (Å)	84.33	133.6
c (Å)	90.53	71.28
Resolution Limits (Å)	84.3–2.2 (2.3–2.2)	49.5–2.4 (2.5–2.4)
Completeness (%)	98.9 (93.2)	97.6 (81.7)
Measured Reflections	145421 (10149)	95760 (3980)
Unique Reflections	16985 (2266)	15750 (933)
Multiplicity	8.6 (4.5)	6.1 (4.3)
R_merge_<I/σ(I)>	0.081 (0.620) 15.2 (2.2)	0.11 (0.67) 10.1 (2.2)

Values in parentheses refer to the outer resolution range.

Japanin-C222_***1***_: a prismatic crystal grew in condition 1 of the JCSG + screen^51^: 0.2 Li_***2***_SO_***4***_, 0.1 M CH_***3***_COON a pH 4.5 and 50% v/v PEG 400. A 2.4 Å X-ray diffraction dataset was collected and indexed in a C-centred orthorhombic space group. Systematic extinctions suggested C222_1_. The same C222_1_ crystal form also grew in condition H5 of the Molecular Dimensions Structure screen 2–41: 0.01 M cetyltrimethylammonium bromide, 0.5 M NaCl and 0.1 M MgCl_2_. This crystal gave 2.5 Å diffraction data and a structure that is equivalent to the one described here.

Diffraction data were collected on beamline ID29 at the ESRF, Grenoble, France. X-ray data integration and scaling were done using the computer programs XDS^52^ and Scala^53^, in the CCP4 suite, run from the data processing suites xia2^54^ and autoPROC^55^. Table 2 shows the crystallographic data collection and processing statistics.

### Phasing and refinement

The Japanin-P4_1_2_1_2 crystal form was phased by molecular replacement with the computer program Phaser^56^ in the CCP4 suite, using a search model obtained from PDB ID 1qft, modified using Chainsaw^57^ in the CCP4 suite and further manual trimming of loops. One copy in the asymmetric unit in P4_**1**_2_**1**_2 gave a good initial hit which was then improved by iterative automated model building in Buccaneer^58^ in the CCP4 suite, using 2Fo-Fc maps computed in autoBUSTER^59^ and alternating cycles of full B refinement and TLS refinement. The program Coot^60^ was then used for manual rebuilding, again using autoBUSTER for refinement. The model for the bound cholesterol was built in the residual electron density (see Fig. 3) once the model for the protein had been completed, starting from the idealised coordinates and stereochemical dictionary downloaded from the Hic-UP server^61^.

The Japanin-C222_1_ crystal form was phased by molecular replacement using the Japanin-P4_1_2_1_2 coordinates as a search model. The program Phaser^56^ in the CCP4 suite placed two molecules in the asymmetric unit of C222_1_. Refinement in autoBUSTER and manual building in Coot followed. All stages of refinement implemented automated non-crystallographic symmetry restraints. Table 3 contains the final crystallographic refinement data and statistics.

**Table 3:** Refinement statistics.

PDB ID	4boe	4bqu
Space Group (Z)	P4_***1***_2_***1***_2 (8)	C222_***1***_ (16)
Resolution range (Å)	61.7–2.2 (2.3–2.2)	49.5–2.4 (2.4–2.5)
Observations	16158 (2771)	15712 (2508)
**Free set**	812 (178)	785 (132)
R	0.1794 (0.1844)	0.199 (0.217)
R_work_	0.178 (0.182)	0.196 (0.215)
R_free_	0.198 (0.223)	0.252 (0.254)
Rmsd bond lengths (Å)	0.01	0.01
Rmsd bond angles (°)	1.05	1.14
Ramachandran outliers	0	1
Ramachandran favoured	99.33%	95.40%
Residues modelled (range)	1–154	A:2–152; B:2–150
Waters modelled	89	151
Average B (protein)	46	50.3
Average B (water)	53	53.3
Non-protein molecules	5 MPD, 3 imidazole, 1 cholesterol	7 ethylene glycol, 2 cholesterol

Values in parentheses refer to the outer resolution range.

The diffraction data and coordinates have been deposited as PDB IDs 4boe and 4bqu for the tetragonal and orthorhombic crystal forms respectively.

### Multiangle Laser Light Scattering

Size exclusion chromatography was performed on a Superdex200 10/30 column (GE Healthcare) equilibrated in 50 mM Tris.HCl, pH 7.5, 150 mM NaCl at 0.4 ml/min. The column was followed in-line by a Dawn Heleos-II light scattering detector (Wyatt Technologies) and an Optilab-Rex refractive index monitor (Wyatt Technologies). Molecular mass calculations were performed using ASTRA 5.3.4.14 (Wyatt Technologies) assuming a dn/dc value of 0.186 ml/g.

### Data availability

The datasets generated during and/or analysed during the current study are available from the corresponding author on reasonable request.
